# Relationship between ground reaction force, landing kinematics of the ankle, knee, and hip joints, and lower back strength in university-level female netball players

**DOI:** 10.17159/2078-516X/2025/v37i1a21020

**Published:** 2025-08-15

**Authors:** R Janse van Rensburg, HV Hammill, Y Willemse, M Kramer

**Affiliations:** 1 Physical Activity, Sport and Recreation (PhASRec), Faculty of Health Sciences, North-West University (NWU), Potchefstroom, South Africa; 2 Centre for Health and Human Performance (CHHP), Faculty of Health Sciences, North-West University (NWU), Potchefstroom, South Africa

**Keywords:** multi-directional landings, BLC strength, landing kinematics, lower extremity

## Abstract

**Background:**

Netball is a physical, high-intensity, team sport of high concentration movements such as jumping, landing, passing, and catching. The two most common mechanisms of injury in female netball tend to be from high ground reaction forces (GRF) coupled with an incorrect landing technique. The landing technique employed in netball is furthermore influenced by lower back strength (LBS).

**Objectives:**

To assess mean differences in ankle, knee, and hip joint kinematics and ranges of motion between dominant and non-dominant limbs across different landing directions, and secondly, to evaluate the relationships between GRF and LBS in different landing directions during one season in university-level female netball players.

**Methods:**

A cross-sectional, repeated-measures design was employed for this study. University-level female netball players (n=11) were recruited for this study. The back-leg-chest (BLC) dynamometer was used to measure LBS. Different landing kinematics were analysed using a motion capture and force plate system to collect kinematic GRF data, respectively.

**Results:**

Negative (r=−0.01 to −0.51) and positive (r=0.02 to 0.43) correlations were found between GRF and joint ROM. Additionally, negative correlations (r=−0.02 to −0.18) and positive correlations (r=0.00 to 0.27) were found between GRF and BLC strength. Furthermore, negative correlations were found between joint ROM and BLC strength (r=−0.11 to −0.70).

**Conclusion:**

The negative correlations found between joint ROM and BLC strength indicate that individuals with greater BLC strength require less joint ROM during multi-directional landing in elite female netball players. Furthermore, the positive and negative correlations found between GRF and BLC strength are only weak due to significant variability between participants. However, this information still highlights the importance of multi-directional landing within elite female netball players.

Netball is a physically demanding team sport that requires high levels of speed, strength, power, endurance and flexibility.^[1,2]^ In addition, netball is considered to be one of the most popular team sports globally, with literature reporting approximately 20 million male and female elite and non-elite players partaking in the sport.^[3]^ Jumping and landing are essential in netball, with approximately 58 multi-directional jumps per match (~1 per player per minute), yet joint-specific differences across jump-landing directions are not well understood.^[3–5]^ Matches across almost all levels are played intermittently within a 60-minute period divided into four quarters consisting of 15 minutes per quarter.^[3,5,6]^ Although an important skillset within netball is related to jumping and landing, with approximately 58 multidirectional jumps being recorded in typical match scenarios, which is almost one per player per minute, it is not clear whether joint-specific differences are present across different jump-landing directions.^[5,7,8]^

Ground reaction forces (GRF) refer to the forces that are exerted by the ground on the player when the player’s body makes contact, especially during jumping and landing, where forces can be several multiples of the player’s body mass.^[8,9,10]^ Moreover, GRFs provide important information related to the motor control associated with jumping-related movements and potential injury risk.^[11]^ Of special importance are the vertical ground reaction force (VGRF) which can range from 3.9–4.3 times a person’s body mass,^[8,12]^ and can be as high as 6.8 times a person’s body mass,^[18]^ when landing, whereas horizontal ground reaction force (HGRF) ranges from 4.2–4.6 times a person’s body mass when landing.^[8]^ Importantly, the range of motion (ROM) through which the lower extremities move during landing can mitigate higher landing forces by increasing the time over which the centre of mass is decelerated.^[13]^ The extent to which joint-specific ROM changes as a function of landing direction has received limited attention, thereby warranting further investigation.

Landing technique can play an important role, whereby a netball-specific landing techniques consist of a ground contact phase and a deceleration phase.^[14]^ The ground contact phase encompasses initial ground contact, unilateral- or bilateral landings, foot placement, and stance width.^[20]^ The deceleration phase comprises the landing foot angle, knee motion, multi-joint flexion, and core angle at landing.^[14]^ The landing technique employed directly impacts the capacity of the joints to absorb energy associated with high GRF during landing.^[14]^ Optimal landing kinematics (such as a soft landing style) can be defined as optimal lower extremity flexion of the ankle, knee, and hip joints; the shoulders and hips should also be aligned with the chest over the knees when landing.^[8]^ Lower extremity ROM and muscle activity, along with deceleration on the penultimate step before catching the ball, help distribute GRF more evenly. This distribution occurs both anatomically across structures and temporally over a longer period. As a result, peak GRF is reduced, leading to more optimal lower extremity landing kinematics.^[15]^

Additionally, the literature states that players should aim to implement a soft-landing technique in diagonal- and lateral directions for both the dominant and non-dominant limb as it potentially lowers the risk for lower extremity injuries.^[14]^ Injury prevalence tends to be high in netball due to the sport’s popularity and physical nature, with the ankle joint accounting for the most injuries, followed by the knee joint. Injuries to the lower back region are identified as the third most common area of injury in elite female team sports.^[2,9]^ Therefore, knowledge of the relative joint ROM requirements and their relationship to lower back strength (LBS) during landing across multiple directions may facilitate and guide safe and effective training practices.

Guarding against injuries to the lower back region and thereby preventing lower back pain (LBP) can be achieved by optimal lumbo-pelvic-hip complex (LPHC) stability.^[15]^ LPHC stability is achieved by optimal lower back strength (LBS) and core activation, since optimal core strength plays a key role in stabilising the spine and provides protection against LBP and influences physical performance.^[15]^ Dynamic postural control can be defined as performing a functional task with strength and purposeful movements (i.e., jumping and landing) that translate the body’s centre of mass whilst maintaining a stable base. Literature has also shown that optimal neuromuscular activation, achieved through a warm-up that includes knee stability, landing techniques, and core stability, can decrease knee injuries by 64%^[15]^, highlighting the importance of core stability in movement patterns. However, it is presently unclear whether landing techniques change appreciably across multiple directions, nor whether the overall strength of the lower back is a contributing factor, thereby presenting a gap in the current literature.

The high intensity and multi-directional jump landings within netball justify the evaluation of LBS, GRF, and landing kinematics of the ankle, knee, and hip joint to assess factors that can predispose netball players to lower back injuries, LBP, and lower extremity injuries. The value gained from this study will enable coaches to focus on these aspects, reducing the incidence of selected injuries in netball and enhancing physical performance. The objectives of this article are, firstly, to evaluate the relationships between GRF and the kinematics of the ankle, knee, and hip joints in different landing directions, and secondly, to evaluate the relationships between GRF and LBS across different landing directions in university-level female netball players during one season.

## Methods

### Study design

For the study a cross-sectional, repeated measures design was implemented.

### Participant selection

The planned sample size of (n=13) participants for the study was calculated *a priori* using the following input parameters for a within-group repeated measures analysis of variance: (i) anticipated effect size (f)=0.25, (ii) type-1 error rate=0.05, (iii) type-2 error rate=0.20, (iv) number of measurements=5, (v) expected correlation of at least 0.70.^[12]^ Due to the implications of COVID-19 and the high physical demands of netball, the final sample was eleven (n=11) university-level female netball players. The age range for the university-level female netball players recruited to participate voluntarily in the study is between 19 and 25 years.

### Ethical considerations

Approval to test NWU female netball players was obtained from the Research Data Gatekeeper Committee (RDGC) (NWU-GK-21-070). Additionally, ethical approval for the study was obtained from the Health Research Ethics Committee (HREC) at NWU (NWU-00195-21-S1).

### Lower back strength (LBS)

The maximal isometric force of the muscles connected to the lower back muscle group (lumbar spine, pelvis, and hip) was tested using the back-leg-chest (BLC) dynamometer (Omron, Kyoto, Japan). The BLC dynamometer has a high test-retest reproducibility, with r values of 0.92 in females and 0.93 in males, and is often used in research to validate strength training techniques. Participants were familiarised with the BLC dynamometer through one trial repetition each. Before the trial repetition, the correct testing position was ensured by adjusting the chain length for each participant according to their height. This was accomplished by asking the participants to stand with both feet on the base of the BLC dynamometer, with their knees and hips slightly flexed whilst maintaining a normal lordotic curve. The handle of the BLC dynamometer was positioned at the height of the intra-articular surface of the knee joint whilst the participants were standing upright at the base of the BLC dynamometer with knees extended. Instruction was then given to provide maximal isometric contractions of the lower back muscle group, knees, and hip by performing a torso extension gradually in a vertical motion, holding the contraction for three to five seconds. This was repeated until three successful repetitions were recorded for each participant with a 60-second rest period between repetitions. The highest value was recorded in kg and retained for analysis.

### Landing kinetics and kinematics of the ankle, knee, and hip joint

**Fig. 1:**
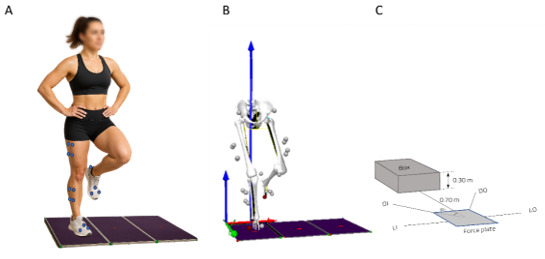
Experimental setup. Panel A shows a representative athlete during the unilateral jump landing on the dominant limb. Panel B shows the processed data from the same athlete for the kinetic and kinematic data extraction. Panel C shows the dimensions, orientation, placement of the box in relation to the centre of the force plate and where each jump would be initiated from.

Figure 1 shows the experimental set up for the landing kinetics and kinematics. Before jump testing, all participants completed a warm-up consisting of three minutes on a stationary bike with the resistance set to two and revolutions per minute set to 60 (approximately 120 watts). Dynamic stretching routines of the following muscle groups were completed after the warm-up: hamstrings, quadriceps, calves, glutes, and hip flexors. Familiarisation with the test procedure was ensured by giving each participant two warm-up jumps in each direction. Testing was done with shoes to mimic netball matches and training scenarios closely.

All jump-landing tasks were evaluated using a motion capture system (Qualisys Medical AB, Gothenburg, Sweden) to collect kinematic data at 200 Hz. A total of eight near-infrared cameras were used (Oqus 7+ series, Qualisys, Sweden), which were synchronised with force plates (AMTI, BMS400600, Watertown, MA, USA), recording GRF at 2000 Hz. Calibration of all eight cameras took place before data collection. Kinematic data were collected by placing 23 retro-reflective markers on the following landmarks: anterior-superior-iliac-spines (ASIS), anterior thighs, greater trochanters, medial- and lateral femoral epicondyles, anterior shanks, medial- and lateral malleoli, calcaneus and the first and fifth metatarsal heads, with one marker on the sacrum. The single leg jumps took place from a 30 cm high platform located 0.7 metres (m) from the centre of the force plate to standardise the landing height and distance. The following landing directions were tested: straight jump (SJ), diagonal inside (DI), diagonal outside (DO), lateral inside (LI), and lateral outside (LO), with each direction oriented at a 45-degree angle relative to the others.^[23]^ Each participant was required to complete five successful unilateral jumps in each direction, on both the dominant and non-dominant leg (i.e., a total of 50 jumps; 25 per limb). A successful trial was retained for analysis upon meeting the following criteria: (i) the participant must face forward, (ii) the participant should initially land with fully extended knees, followed by joint flexion for shock absorption, and (iii) hands should remain on the waist. Unsuccessful trials were excluded, and trials were repeated until five successful trials occurred in each direction on both the dominant and non-dominant leg. Participants rested for 30 seconds between jumps in the same direction for each leg and two minutes for jumps in different directions.

A fourth-order zero-lag Butterworth filter was used to process the raw marker data, with cutoff frequencies set at 6 Hz to 15 Hz for individual markers. A 50 Hz cutoff was used for the force plate data, following detailed residual analyses and visual inspection of the data. Kinematic data was then processed using Visual 3D (C-Motion Inc., Gaithersburg, USA) to obtain the relevant joint angles. The 3D kinematics of the hip, knee, and ankle were evaluated according to a XYZ Cardan sequence of rotations (X is flexion-extension, Y is abduction-adduction, Z is internal-external rotations). During the landing phase, the force waveform data was normalised to 101 points to standardise the movement times of all participants.

### Statistical analyses

All statistical analyses were completed using JASP (JASP Team, version 0.18.1, Netherlands) and the R programming language (Version 2022.04.01, RStudio, Posit Software PBC, URL: https://posit.co/download/rstudio-desktop/) (R Core Team, 2023). The Shapiro-Wilk test was used to assess whether the data were normally distributed, and normality was accepted when the p-value exceeded the alpha threshold of 0.05. To evaluate whether mean differences in landing kinematics of the ankle-, knee-, and hip joints were significantly different between limbs (i.e., dominant vs. non-dominant) across each landing direction (i.e., 5-levels), a linear mixed effects model was used where the landing direction served as the fixed effect and participants served as the random effect. *Post-hoc* analyses were conducted to evaluate pairwise contrasts for each limb and landing direction, where a Holm correction was implemented to minimise the type-1 error rate, and Cohen’s d served as a measure of the standardised effect size. The magnitude of Cohen’s d was qualitatively interpreted as: trivial (<0.10), very small (0.10–0.19), small (0.20–0.49), medium (0.50–0.79), large (0.80–1.19), and very large (>1.20). A correlation analysis between mean ankle-, knee-, and hip ROM, and peak landing forces and BLC was completed using Spearman’s rank correlation (r) analysis with a Holm correction to adjust for multiple comparisons. The coefficients are qualitatively interpreted in the following terms: negligible (r=0.00 to 0.10); weak (r=0.11 to 0.39); moderate (r=0.40 to 0.69); strong (r=0.70 to 0.89), and very strong (r=0.90 to 1.0).

## Results

**Fig. 2:**
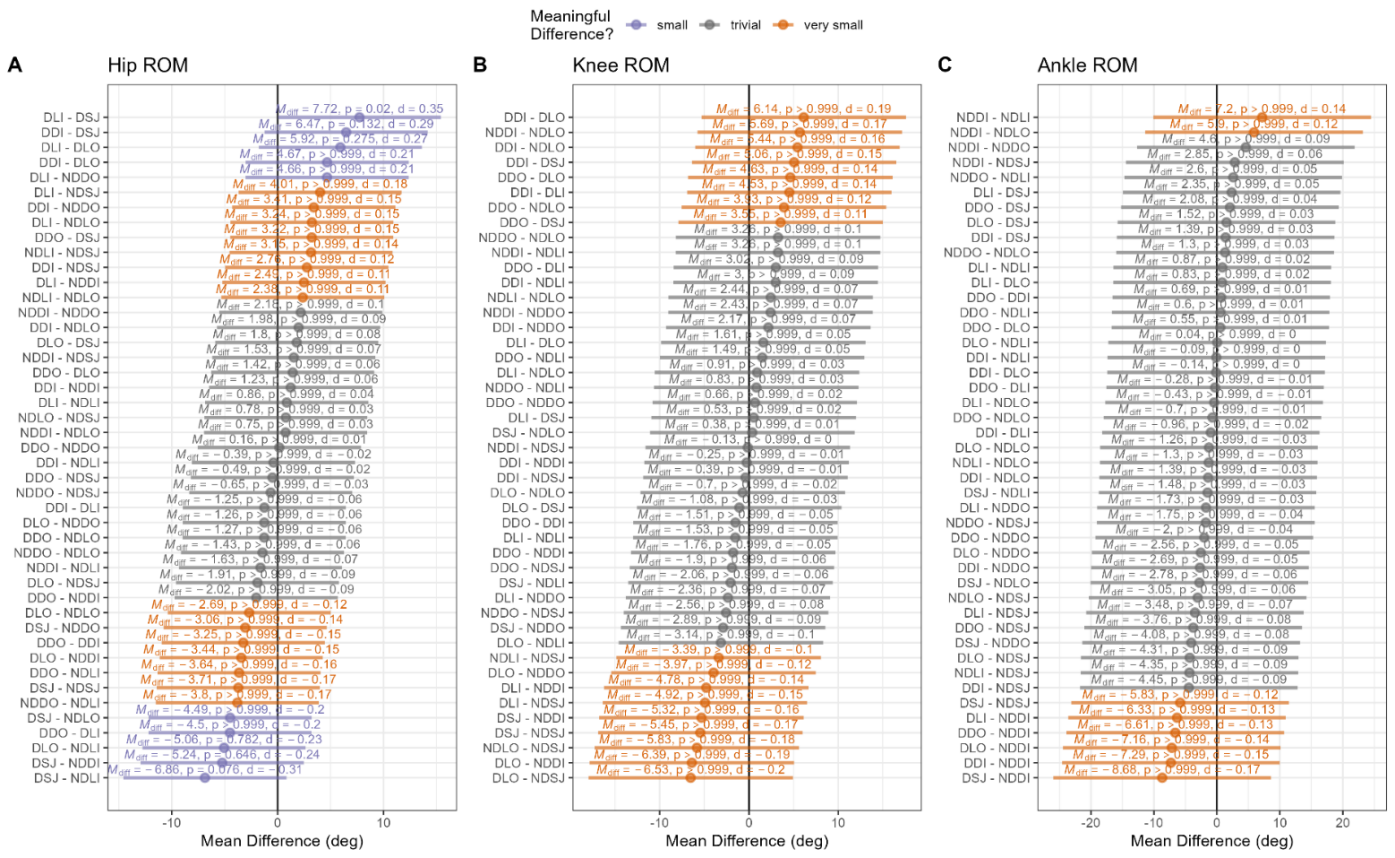
Differences in joint-specific landing kinematics of the hip, knee, and ankle joints across different landing directions. NDSJ, non-dominant straight jump; NDDI, non-dominant diagonal inside; NDDO, non-dominant diagonal outside; NDLI, non-dominant lateral inside; NDLO, non-dominant lateral outside; DSL, dominant straight jump; DDO, dominant diagonal outside; DDI, dominant diagonal inside; DLO, dominant lateral outside; DLI, dominant lateral inside.

The results of the mean differences in joint-specific ROM across each limb and landing direction are highlighted in Figure 2.

Sagittal plane kinematics of the ankle, knee, and hip joints, along with VGRF data, were collected to achieve the objectives of the present study. Although differences in joint ROM are present between limbs and across multiple directions, these are generally less than 10 degrees. Moreover, there is considerable variability between the ROM required during multi-directional landing, as indicated by the width of the error bars. Subsequently, there is inconclusive evidence that landing limb or landing direction has a meaningful influence on the joint ROM required for optimal landing within elite-level female netball players.

**Fig. 3:**
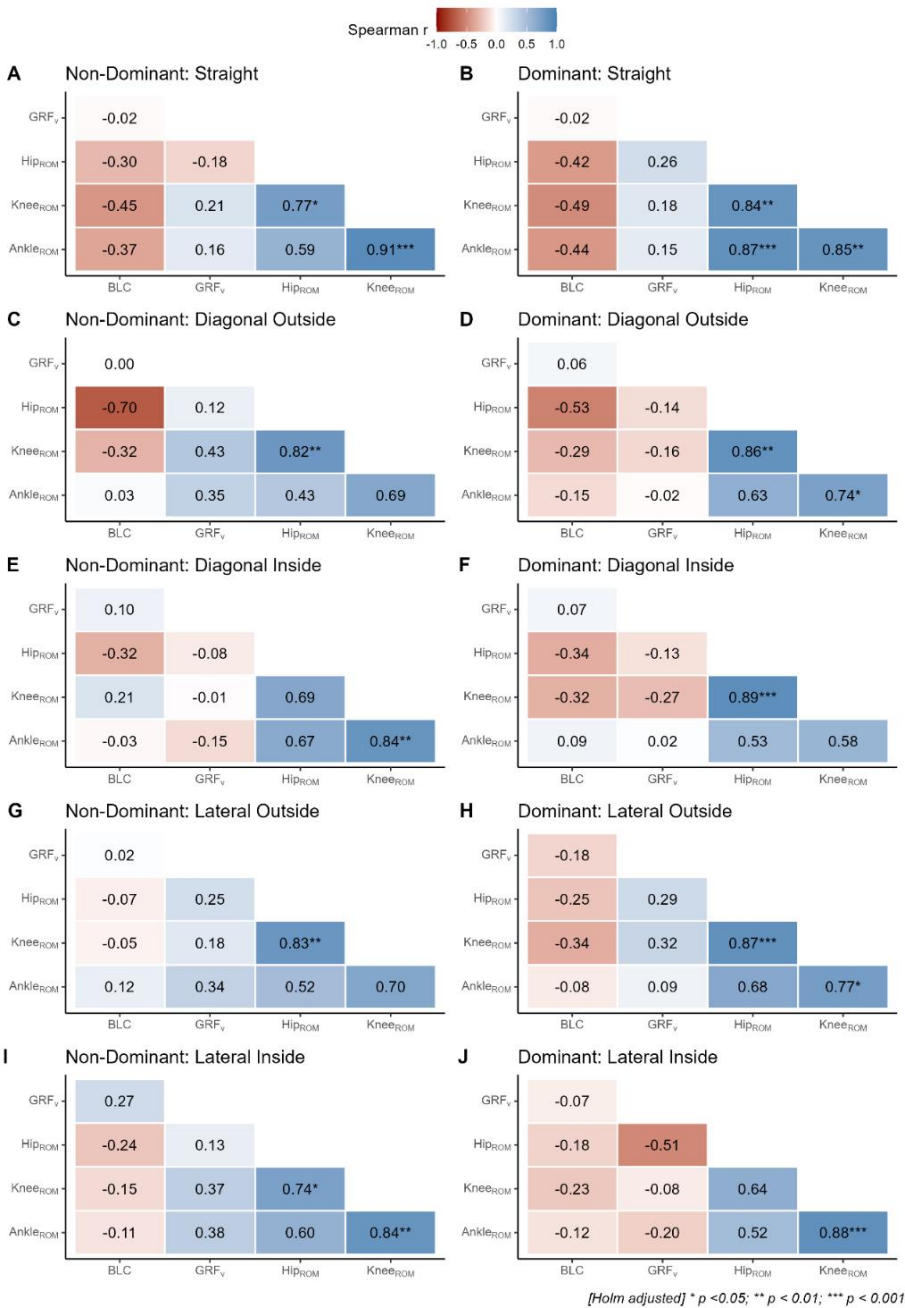
Correlations between BLC strength, GRF, and kinematics of the ankle, knee, and hip joint for the dominant and non-dominant limb in multi-directional landing. ROM, range of motion; VGRF, vertical ground reaction force; BLC, back-leg-chest strength.

The associations between limb kinematics, VGRF, and LBS for each limb and jump-landing direction are shown in Figure 3.

There are weak-to-strong negative correlations between joint ROM and BLC strength (r=−0.11 to −0.70). This implies that individuals with greater BLC strength will typically experience lower joint ROM requirements (or those with lower BLC strength require higher joint ROM) during multi-directional unilateral landing. Such an interpretation appears to be even more pertinent in the dominant limb as compared to the non-dominant limb of female netball players.

Furthermore, a negligible-to-moderate negative (r=−0.01 to −0.51) and negligible-to-moderate positive (0.02 to 0.43) association can be seen between joint ROM and GRF. Overall, individuals with higher ROM also exhibited higher landing forces; however, the evidence for this is weak due to substantial variability among participants.

Additionally, there are moderate to strong positive correlations between the ankle and hip ROM (r=0.43 to 0.87). Moderate to strong positive correlations can also be observed between the knee and hip ROM (r=0.64 to 0.89). Moreover, there are moderate to very strong positive correlations between the ankle and knee ROM (r=0.58 to 0.91). Furthermore, negligible to weak (r=0.00 to 0.27) positive and negligible to weak (r=−0.02 to −0.18) negative correlations can be found between GRF and BLC strength. The evidence supporting this is weak due to substantial variability between participants.

## Discussion

The primary objectives of this study were to evaluate the differences in the joint-specific kinematics of the ankle, knee, and hip joints within different landing directions, and secondly, to evaluate the relationships between joint kinematics, GRF, and LBS across different landing directions.

Our results showed that although there were differences in joint ROM present between the dominant and non-dominant limb across multi-directional landings, these were regularly less than 10 degrees. A recent meta-analysis comparing individuals with anterior cruciate ligament injuries to their healthy contralateral limbs and healthy controls revealed that hip, knee, and ankle angles of ~5.1°, 5.1°, and 4.7°, respectively, represented meaningful differences during jump-landing tasks. Within this context, our results indicate that small yet meaningful differences exist in the landing kinematics between limbs and landing directions for each of the major joints, which may need to be considered by coaches and practitioners (Figure 2). Whether such differences pose a risk for future injury is not clear and would support the need for future research comparing healthy with compromised groups. The relative consistency of joint kinematics across limbs and jumping directions may likely be explained by the notion that elite athletes typically develop highly refined and consistent movement patterns through extensive training. Their neuromuscular control may minimise differences in joint ROM regardless of which limb they use or the direction of landing. Landing biomechanics often show that joint ROM remains relatively stable across different landing conditions in highly trained individuals. This stability may be due to optimised neuromuscular strategies that allow them to adapt efficiently to different landing scenarios. Elite netball players may unconsciously adjust other kinematic variables (e.g., trunk positioning, knee flexion angles, or muscle activation patterns) to maintain consistent joint ROM, reducing the impact of landing limb or direction on overall landing mechanics.

Data from a previous study on 10 junior male volleyball players^[16]^, further supports our results. This study showed a lack of evidence to support a relationship between the lower extremity kinematics of the ankle, knee, and hip joints in unloaded vertical jump-landings as compared to loaded vertical jump-landings. No significant differences were noted between unloaded- and loaded landings with peak ankle (p=0.370), knee (p=0.501) and hip (p=0.594) flexion moments observed to be similar.^[16]^ Additionally, landing kinematics in four different directions (forward, 30° diagonal, 60° diagonal, and lateral) were evaluated in 18 male athletes, comprising nine basketball players and nine volleyball players, with increased knee flexion observed. In contrast, hip flexion decreased at initial contact.^[17]^ Moreover, during landing, individuals showed altered peak ankle dorsiflexion angles, plantar flexor moments, as well as modified hip flexion angles that were sensitive to the landing direction.^[17]^

Our study, in conjunction with the literature, therefore supports the conclusion that landing limb or landing direction tends to exhibit small yet meaningful influences on the joint ROM required for landing in elite female netball players. These findings are reinforced by the moderate-to-strong positive correlations between the ankle and hip ROM (r=0.43 to 0.87) during multi-directional landings. This would imply that individuals with greater ankle ROM will typically experience greater hip ROM during multi-directional landing for the dominant and non-dominant limb in elite-level female netball players, given the substantial contribution of the ankle during force absorption while completing single-leg landing tasks. Furthermore, moderate-to-strong positive correlations were observed between the knee- and hip ROM (r=0.64 to 0.89), which is also explained by the joint contributions evident during horizontal jumping (reference). This implies that individuals with greater knee ROM will typically experience greater hip ROM during multi-directional landing for the dominant and non-dominant limb in elite-level female netball players. It was furthermore observed that there were moderate-to-very strong positive correlations between the ankle and knee ROM (r=0.58 to 0.91) across different landing directions. The implication here is that individuals with greater ankle ROM will typically experience greater knee ROM during multi-directional landing for the dominant and non-dominant limb in elite-level female netball players. Given the multi-joint nature of jump-landing, positive correlations between the ROM of load-bearing joints such as the ankle, knee, and hip are not surprising. Of greater interest is the variable association of joint-specific ROM and VGRF, which was positive but weak-to-moderate for some directions (r=0.16 to 0.43; NDSJ, NDDO, NDLO, DLO), whereas it was negative and weak-to-moderate for other directions (r=−0.13 to −0.51; DDI, DLI, NDDI). This suggests that, at least within the evaluated sample, joint stiffness (i.e., harder vs. softer landings) can attenuate landing forces, and these changes considerably depending on both the landing limb and direction. The extent to which this is malleable through training and the potential effects on injury risk would, however, require further research. These latter interpretations are supported by the study, which evaluated the landing kinematics of 21 skilled female netball players and found significant correlations between ankle ROM when landing on the non-dominant limb and VGRF (r=−0.648)^[7]^. According to literature, forefoot- and heel landings further influence VGRF generated in landing kinematics in elite female netball players, as research shows mean peak VGRF for single leg landings to be 5.3 times an individual’s body mass, whilst heel landings represent 5.7 times an individual’s body mass.^[13]^ Research further states that soft landings will reduce GRF, as during a soft landing, muscles can absorb 19% more energy when compared to hard landings.^[18]^ Although this was not tested in the current study, further investigation should clarify the aforementioned mechanism, which may offer a strategy that can decrease GRF.

Regarding the role of whole-body strength during landing, the present study found negligible-to-weak positive correlations (r=0.00 to 0.27) and negligible-to-weak negative correlations (r=−0.02 to −0.18) between GRF and BLC strength across different landing directions. Research has shown that an increase in strength can enhance an individual’s ability to maintain static and dynamic positions.^[10]^ Furthermore, studies have shown that a strong relationship exists between strength and GRF where those individuals with greater strength produce higher propulsive GRF and impulses during explosive movements such as jumping, sprinting, and direction changes.^[10]^ Whether greater strength plays a more protective role in force attenuation and injury risk would require longitudinal research, which is currently lacking, especially within a South African context. Lastly, the associations between joint ROM and BLC strength indicated weak-to-strong negative correlations during multi-directional landings (r=−0.11 to −0.70). This likely implies that individuals with greater BLC strength will have lower ROM requirements to control and stabilise the centre-of-mass during multi-directional landing. Previous research has shown that ankle inversion and eversion strength, as well as knee flexion and extension strength, are significant predictors of dynamic postural stability. According to our research, this effect appears to be more pronounced in the dominant limb compared to the non-dominant limb of elite female netball players. The findings from the current study are supported by Wei *et al.*^[19]^ who conducted a study on 18 elite skiing individuals and found that optimal LBS contributed to more stable landings due to a reduction in the hip- and knee flexion required, which further reduced the GRF during impact. Additionally, research indicates that LBS contributes to improved overall physical health and enhanced physical performance by achieving LPHC stability, which in turn improves core stability and dynamic postural control.^[14,20]^ Furthermore, a study from Barnes *et al.*^[20]^ showed that elite university-level female netball players in South Africa tend to have poor dynamic postural control, emphasising the importance of optimal LBS in dynamic movement patterns. The current study may indicate that strength and conditioning programs for university-level female netball players in South Africa may not sufficiently target LBS development. The sport involves frequent sudden stops, jumps, and directional changes, which require exceptional postural control. Without adequate training, athletes may struggle with maintaining stability during multidirectional landing tasks. Differences in training facilities, coaching techniques, and exposure to strength and conditioning programs can influence the development of dynamic postural control. Whether these factors are truly causal would, however, require further research in the form of well-designed randomised control trials.

Possible limitations of the present study include that only university-level female netball players were studied, and that the sample was small, with a limited number of participants (n=11). Furthermore, the study focused not only on overall LBS (lumbar spine, pelvis, and hip) outcomes but also on specific muscles of the lumbar spine. The same study should be replicated with a larger sample size across a broader range of playing abilities. Due to the small sample size of elite university-level female netball players, the generalisability of the findings should be treated with some caution. The study should also not be generalised to other sporting codes. Finally, future studies should analyse lower limb muscle activity to determine if reduced ankle, knee and hip ROM from increased LBS is offset by greater eccentric strength in these joints.

## Conclusion

In this study, the kinematics of the ankle, knee, and hip joints were measured in the sagittal plane while evaluating the influence of joint-specific ROM and BLC strength on VGRF. Small but meaningful differences were evident between ankle, knee, and hip ROM of the dominant and non-dominant limb across multiple directions, which may have implications for training and injury risk that need to be considered by coaches and practitioners.

Weak-to-strong correlations between ankle, knee, and hip ROM and BLC strength (r=0.11 to −0.70) were present, indicating that those with greater BLC strength required lower joint-specific ROM to stabilise the centre-of-mass during multidirectional landings.

Lastly, associations between BLC strength and VGRF magnitudes were highly variable, indicating that the landing limb and direction are confounding factors during force attenuation.
